# Alteration of brain nuclei in obese children with and without Prader-Willi syndrome

**DOI:** 10.3389/fninf.2022.1032636

**Published:** 2022-11-18

**Authors:** Ning Wu, Huan Yu, Mingze Xu

**Affiliations:** ^1^Department of Medical Imaging, Yanjing Medical College, Capital Medical University, Beijing, China; ^2^Department of Radiology, Liangxiang Hospital, Beijing, China; ^3^Center for MRI Research, Academy for Advanced Interdisciplinary Studies, Peking University, Beijing, China

**Keywords:** childhood obesity, genetic imprinting, Prader-Willi syndrome, brain nuclei, shape analysis, hypothalamus, deep cerebellar nuclei

## Abstract

**Introduction:** Prader-Willi syndrome (PWS) is a multisystem genetic imprinting disorder mainly characterized by hyperphagia and childhood obesity. Extensive structural alterations are expected in PWS patients, and their influence on brain nuclei should be early and profound. To date, few studies have investigated brain nuclei in children with PWS, although functional and structural alterations of the cortex have been reported widely.

**Methods:** In the current study, we used T1-weighted magnetic resonance imaging to investigate alterations in brain nuclei by three automated analysis methods: shape analysis to evaluate the shape of 14 cerebral nuclei (bilateral thalamus, caudate, putamen, globus pallidus, hippocampus, amygdala, and nucleus accumbens), automated segmentation methods integrated in Freesurfer 7.2.0 to investigate the volume of hypothalamic subregions, and region of interest-based analysis to investigate the volume of deep cerebellar nuclei (DCN). Twelve age- and sex-matched children with PWS, 18 obese children without PWS (OB) and 18 healthy controls participated in this study.

**Results:** Compared with control and OB individuals, the PWS group exhibited significant atrophy in the bilateral thalamus, pallidum, hippocampus, amygdala, nucleus accumbens, right caudate, bilateral hypothalamus (left anterior-inferior, bilateral posterior, and bilateral tubular inferior subunits) and bilateral DCN (dentate, interposed, and fastigial nuclei), whereas no significant difference was found between the OB and control groups.

**Discussion:** Based on our evidence, we suggested that alterations in brain nuclei influenced by imprinted genes were associated with clinical manifestations of PWS, such as eating disorders, cognitive disability and endocrine abnormalities, which were distinct from the neural mechanisms of obese children.

## Introduction

The pediatric obesity rate has increased explosively over the past 30 years, reaching up to 10% worldwide (Gerhart et al., 2022). This is a critical public health issue, since obesity is associated with far-reaching and costly implications, such as type II diabetes and heart disease (Flegal et al., 2010). Brain circuitry involved in motivation, reward, and cognitive control has been identified to explore neurobiological underpinnings of childhood obesity (Bruce et al., 2010).

Prader-Willi Syndrome (PWS) is a typical genetic obesity disease caused by the loss of paternal gene expression located in chromosome 15q11-q13 (Bittel and Butler, 2005). It is characterized by variable and typical phenotypes, affecting one in 15,000–20,000 live births (Soni et al., 2008). The predominant clinical manifestations of PWS are hypotonia and poor suck before the neonatal stage; as age increases, these transition to developmental delay and an insatiable appetite, showing hyperphagia as well as morbid and even life-threatening obesity without external restriction of food intake (Butler et al., 2006; Angulo et al., 2015). In addition, other phenotypes, such as hypogonadism, temperature instability, high pain threshold, hypersomnia, mild to moderate intellectual disability, and avariable range of social and behavioral difficulties, and psychiatric disturbances also occur in children with PWS (Dykens and Kasari, 1997; Clarke et al., 2002; Holland et al., 2003).

These diverse symptoms indicate a widespread developmental abnormality in the brain structure of children with PWS. Eating behavior is known to be regulated by high levels of cognitive control and satiety mechanisms based on brain subcortical reward and motivational neurocircuitry involving the amygdala, hippocampus, caudate nucleus, putamen, pallidum, nucleus accumbens (NAc), septal nucleus and thalamus (Orsi et al., 2011; Kirouac, 2015). As the predominant endocrine gland, the hypothalamus is responsible for sensing nutrients and regulating neuropeptides such as leptin, ghrelin, and insulin (Volkow et al., 2011; Angulo et al., 2015). Additionally, deep cerebellar nuclei (DCN) are associated with motor control, hypotonia, and satiation networks for sensing nutrient infusion in the gut and releasing dopamine (Blanco-Hinojo et al., 2021; Low et al., 2021). All of these brain nuclei comprehensively contribute to the recognition and regulation of homeostatic and hedonic eating behavior.

Previous neuroimaging studies of PWS have focused on cortical reward and inhibitory circuities and found functional deficits in multiple related regions, such as the dorsolateral and medial prefrontal cortex, orbitofrontal cortex, right anterior cingulate cortex, temporal lobe, and insula (Holsen et al., 2006; Xu et al., 2017). However, only a few studies have paid attention to the alteration of brain nuclei, let alone in children with PWS (Honea et al., 2012; Xu et al., 2017; Manning et al., 2018). Honea et al. (2012) found lower gray matter volume (GMV) in the right hippocampus in young adults with PWS. Lukoshe et al. (2017) reported no volumetric differences in the hypothalamus and mammillary bodies in children with PWS. In cerebellar studies, decreased whole cerebellar and relative lobular volume ratios in posterior inferior lobules in PWS individuals compared with normal controls were reported (Honea et al., 2012; Lukoshe et al., 2013, 2017; Yamada et al., 2020). In conclusion, due to the limited number of cerebral and cerebellar nuclei investigated in previous findings, a systematic and comprehensive study of the brain nuclei in children with PWS is needed.

To date, segmentation methods of subcortical nuclei for PWS have been concentrated on manual segmentation and voxel-based morphometry (VBM). However, there are still several knowledge gaps. First, based on voxel-wise comparison, VBM is prone to registration artifacts in the deep gray matter and may not be suitable for the analysis of cerebral nuclei. Second, arbitrary smoothing makes VBM insensitive to boundary location, and it is difficult to detect subtle atrophy of nuclei. Third, relatively small studies such as those involving PWS run the risk of false-positive findings or nonsignificant results using hypothesis-free segmentation methods (Patenaude et al., 2011; van den Bogaard et al., 2011; Nemmi et al., 2015; Zonneveld et al., 2019). Subcortical shape analysis can provide a direct, purely local measure of geometric changes by analyzing differences in boundary vertex locations, which has been applied to Parkinson’s disease and Huntington’s disease to find cerebral nuclei atrophy (van den Bogaard et al., 2011; Nemmi et al., 2015). Meanwhile, the shape analysis method has been validated in pediatric studies and makes it a promising technique for cerebral nuclei segmentation in children with PWS (Ortega et al., 2019; Lidauer et al., 2021). Moreover, it is well known that the hypothalamus is an indispensable nucleus in eating control, and because of its small size, the conventional segmentation method relies on manual delineation (Baroncini et al., 2012). A recent study accomplished auto-segmentation of 10 subregions of the hypothalamus by a deep convolutional neural network, which has been verified in several diseases (Billot et al., 2020; Chao et al., 2022; Lee et al., 2022), so the method was used in our article to investigate alterations in the hypothalamic subunits of PWS and OB subjects.

**Figure 1:**
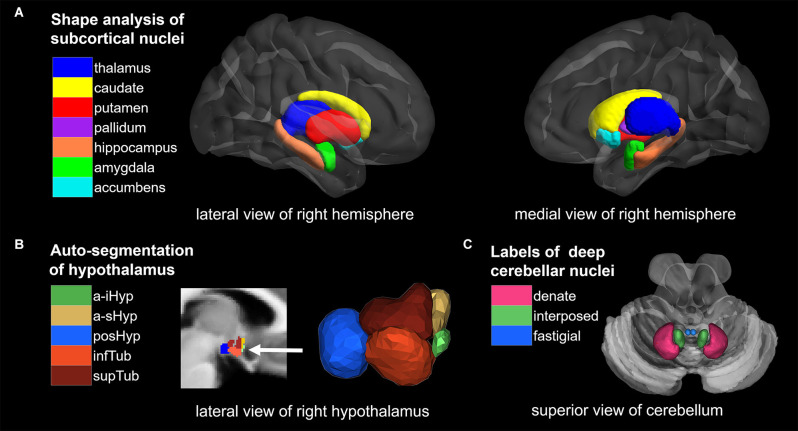
Overview of segmentation methods and corresponding brain nuclei. **(A)** Shape analysis of subcortical nuclei. **(B)** Auto-segmentation of hypothalamus. **(C)** ROI analysis of deep cerebellar nuclei (DCN). NAc, nucleus accumbens; a-iHyp, anterior-inferior; a-sHyp, anterior-superior; posHyp, posterior; infTub, inferior tubular; supTub, superior tubular.

In summary, children with PWS and obesity share similar alterations in the cerebral cortex, which are mainly caused by acquired plasticity of the brain, while alterations in brain nuclei are closely related to inheritance (Roshchupkin et al., 2016). The current study aims to analyze the differences in brain nuclei between PWS, OB, and control children through multiple convincing analysis methods to reveal neural mechanisms involved in extreme hyperphagia and dietary restraint in PWS and obesity. We hypothesized that children with PWS would exhibit genetically influenced impairment in brain nuclei compared with the control group, which was not found in children with obesity. To test our hypothesis, we performed high-resolution T1-weighted magnetic resonance imaging (MRI) in a three-group study and implemented a systematic quantitative study on PWS brain nuclei using auto-segmentation techniques (Figure 1), which included shape analysis for cerebral nuclei, automated segmentation approach for subregions of the hypothalamus, and region of interest (ROI)-based analysis for DCN.

## Materials and Methods

The study was approved by the Institutional Review Board at the University of Florida. We certify that all applicable institutional and governmental regulations concerning the ethical use of human volunteers were followed during this research.

### Subjects

A total of 12 children with PWS (eight girls and four boys; age mean 7.2 years), 18 obese children (12 girls and six boys; age mean 9.0 years), and 18 healthy, normal-weight children (12 girls and six boys; age mean 8.3 years) participated in this study. The subjects in this study were the same as those in Xu et al. (2017) and a part of Miller’s (2006, 2007) study. The image data of subjects was authorized by Xu et al. (2017) and was re-used in this study. Written informed consent and child assent were obtained from all participants and guardians before any aspect of the research was initiated. The three groups were age- and sex-matched (*P* > 0.05). All PWS subjects were characterized by DNA methylation analysis at the 5’ SNURF-SNRPN locus and by fluorescence *in situ* hybridization (FISH) using the SNURF-SNRPN probe and a distal chromosome 15 control probe. None of the patients with PWS were currently at the stage of receiving growth hormone therapy or estrogen/androgen replacement therapy when recruited for the MRI scans. All children with obesity were recruited based on a history of a body mass index (BMI) >95% for age and sex before the age of 4 years and had normal chromosomal, DNA methylation, and SNURF-SNRPN FISH analysis for PWS, melanocortin 4-receptor mutation testing, and Fragile X DNA testing. To reduce the effects of socioeconomic and genetic background, siblings of the children with PWS and obesity who were generally closest in age to the probe and who did not have a history of childhood obesity or known genetic abnormalities were recruited in the control group (Miller et al., 2006). Cognitive and achievement testing was performed using the Woodcock-Johnson Test of Cognitive Ability and Academic Achievement-Third Edition (WJ-III; Miller et al., 2006). The body mass index standard deviation score (BMI-z) was calculated from the standardized growth charts distributed by the Centers for Disease Control^1^ (Miller et al., 2006; Xu et al., 2017).

### Image acquisition

All subjects were scanned on a 3.0 T head dedicated Siemens Allegra MRI scanner (Siemens, Munich, Germany). A set of T1-weighted, high-resolution structural images were acquired using a magnetization prepared rapid acquisition gradient echo sequence with the following imaging parameters: matrix size = 512 × 512, field of view = 240 × 240 mm^2^, slice number = 160 slices, slice thickness = 1.1–1.4 mm, TR = 1,500 ms, TE = 4.38 ms, and flip angle = 8°.

### Image processing and measurement

#### Shape analysis of cerebral nuclei

Shape analysis of cerebral nuclei was performed using the FIRST toolbox in FSL, which is a model-based segmentation/registration tool proposed by Patenaude et al. (2011). The FIRST used manually labeled T1-weighted MRI brain image data from 336 normal and pathologic subjects aged from 4.2 to 72 years as a training set, which were provided by the Center for Morphometric Analysis (CrangeMA), MGH, Boston. We began by using FIRST to create vertex meshes for 14 nuclei, including the bilateral thalamus, caudate, putamen, pallidum, hippocampus, amygdala, and bilateral NAc. Each nucleus was parameterized as a surface mesh and then modeled as a point distribution in native space. An example of cerebral nuclei segmentation is shown in Figure 1A, and the quality of segmentations was manually checked by an experienced radiologist. Second, multivariate statistics were performed for each nucleus to yield differences between the groups (PWS vs. OB, PWS vs. control, OB vs. control). The F values and vectors acquired indicate the values and directions of the results, respectively. Outward arrows represent the expansion of the surface, and inward arrows represent atrophy. Finally, correction for multiple comparisons was carried out using false discovery rate (FDR) correction.

In addition, fsl stats were used to obtain the volume of each cerebral nucleus, and volumetric differences among the groups (PWS vs. control, PWS vs. OB, OB vs. control) were calculated with a two-sample *t*-test and FDR correction.

#### Auto-segmentation of hypothalamic subunits

The volumetric analysis was conducted with the HypothalamicSubunits tool integrated in FreeSurfer 7.2.0^2^. This pipeline fulfills automated segmentation of the hypothalamus and its subunits with the deep learning approach of a convolutional neural network (Billot et al., 2020). First, T1-weighted images from all subjects were processed with the FreeSurfer recon-all script. Second, 10 subunits of the hypothalamus in bilateral hemispheres were segmented using the script MRI_segment_hypothalamic_subunits, and the following hypothalamic nuclei were acquired: bilateral anterior-inferior, including the suprachiasmatic nucleus and supraoptic nucleus; bilateral anterior-superior, including the preoptic area and parts of the paraventricular nucleus; bilateral posterior, including the lateral mammillary nucleus, supramammillary nucleus, lateral hypothalamus, and tuberomammillary nucleus; bilateral inferior tubular, including the infundibular nucleus, ventromedial nucleus, and lateral tubular nucleus; and bilateral superior tubular, including the dorsomedial nucleus, lateral hypothalamus nucleus and parts of the paraventricular nucleus. Figure 1B shows the example of a labeled hypothalamic segmentation, and the quality of segmentations was manually checked by an experienced radiologist. Finally, volumetric differences among the groups (PWS vs. control, PWS vs. OB, OB vs. control) were calculated for each hypothalamic subunit with a two-sample *t*-test and FDR correction.

#### ROI analysis of deep cerebellar nuclei

ROI analysis was used to compare the volumes of DCN among groups. First, default VBM processing was conducted with CAT12 (r1907) in SPM^3^, including preprocessing, tissue segmentation, spatial registration to Montreal Neurological Institute (MNI) space and modulation, acquiring modulated and normalized GMV and white matter volume (WMV) tissue maps. In addition, total intracranial volume (TIV) was calculated at this step and was used as a covariate in the subsequent statistics. Second, the volumes of DCN were extracted within the six ROIs from a validated probabilistic atlas of the human cerebellum available in the SUIT (Spatially Unbiased Infratentorial Template) toolbox^4^, which includes the bilateral dentate nucleus, interposed nucleus, and fastigial nucleus (Diedrichsen et al., 2009, 2011). Figure 1C shows the 6 ROIs of the DCN. The sum of total GMV and WMV within the ROI was then measured as the volume of each ROI. Finally, volumetric differences among the groups (PWS vs. control, PWS vs. OB, OB vs. control) were calculated for each ROI with two-sample *t*-test and FDR correction.

### Statistical analysis

All of the numeric statistics, including volumes of cerebral nuclei, hypothalamus subunits, and DCN, were performed with the statistical tool integrated into GRETNA^5^ (Wang et al., 2015). The two-sample *t*-test was performed to examine the significance of the mean volumetric differences among the three groups, with age, sex, and TIV as covariates and P values were corrected for multiple comparisons by FDR (P_FDR_). Results with *P*_FDR_ < 0.05 were considered significant.

The vertex statistics for shape analysis were carried out with the first_utils and were also adjusted for age, sex and TIV. Multiple comparison correction was performed with surface_fdr, and only vertices with *P*_FDR_ < 0.05 were retained.

## Results

### Demographic information

The group comparisons of demographic profile and TIV are listed in Table 1. The BMI-z of the OB group was significantly larger than that of the PWS (*P* = 0.014) and control (*P* < 0.001) groups, and the BMI-z of the PWS group was significantly larger than that of the control (*P* < 0.001). However, it should be noted that BMI underestimates the degree of obesity in PWS due to their decreased muscle mass (Goldstone, 2006). No significant difference was found for age, sex, or TIV between groups.

**Table 1:** Demographic information for the PWS, OB, and control groups.

	**Group**				*P* value		
**Variable**	**Control**	**OB**	**PWS**		**A**	**B**	**C**
Sex (Boys/Girls)	6/12	6/12	4/8		1.000	1.000	1.000
Age (years)	8.3 (0.9)	9.0 (0.9)	7.2 (1.2)		0.466	0.252	0.594
BMI-z	0.79 (0.21)	3.06 (0.19)	2.16 (0.25)		**0.000**	**0.014**	**0.000**
TIV (mm^3^)	1,383.3 (144.5)	1,365.6 (119.6)	1,307.7 (138.0)		0.173	0.238	0.704

Data are presented as the mean (standard deviation) for continuous variables and n/n for sex. Bold, *P* < 0.05. PWS, children with Prader-Willi syndrome; OB, obese children without PWS; BMI-z, body mass index standard deviation score; TIV, total intracranial volume. A: comparison between the PWS and control groups (PWS vs. control). B: Comparison between the PWS and OB groups (PWS vs. OB). C: Comparison between the OB and control groups (OB vs. control).

### Cerebral nuclei

The volumetric analysis of cerebral nuclei investigated the group differences of the whole volume for each nucleus, and the results are listed in Table 2. After controlling for age, sex and TIV, the PWS group showed a significantly decreased volume of the right amygdala (*P*_FDR_ = 0.042) compared with the control and of the bilateral hippocampus [Left (L) and Right (R), *P*_FDR_ = 0.046] and bilateral NAc (L, *P*_FDR_ = 0.042, R, *P*_FDR_ = 0.046) compared with the OB group. No significant results were found between OB and control subjects.

**Table 2:** Cerebral nuclei volumes of each group and *P* value of group comparisons.

	**Mean (SD; mm^3^)**				**Uncorrected *P* value**				***P*_FDR_ value**		
**Nuclei**	**Control**	**OB**	**PWS**		**A**	**B**	**C**		**A**	**B**	**C**
thalamus_L	6,070 (590)	6,496 (919)	5,274 (904)		0.134	0.022	0.219		0.248	0.052	0.352
thalamus_R	6,048 (582)	6,458 (919)	5,274 (890)		0.139	0.023	0.226		0.248	0.052	0.352
caudate_L	2,868 (368)	2,989 (431)	2,603 (471)		0.225	0.122	0.334		0.248	0.131	0.390
caudate_L	3,067 (412)	3,141 (471)	2,567 (486)		0.093	0.055	0.406		0.248	0.086	0.406
putamen_L	3,674 (461)	4,120 (827)	3,484 (643)		0.374	0.073	0.174		0.374	0.102	0.352
putamen_R	3,871 (480)	4,012 (794)	3,390 (683)		0.207	0.088	0.380		0.248	0.110	0.406
pallidum_L	1,281 (129)	1,388 (213)	1,121 (212)		0.170	0.026	0.194		0.248	0.052	0.352
pallidum_R	1,281 (144)	1,393 (223)	1,149 (201)		0.216	0.039	0.201		0.248	0.068	0.352
hippocampus_L	2,513 (348)	2,782 (557)	2,073 (429)		0.136	**0.013**	0.207		0.248	**0.046**	0.352
hippocampus_R	2,448 (397)	2,948 (487)	2,094 (501)		0.180	**0.010**	0.058		0.248	**0.046**	0.352
amygdala_L	815 (95)	852 (128)	746 (108)		0.230	0.094	0.322		0.248	0.110	0.390
amygdala_R	844 (70)	784 (84)	644 (88)		**0.003**	0.261	0.203		**0.042**	0.261	0.352
NAc_L	377 (72)	449 (78)	293 (61)		0.070	**0.003**	0.088		0.248	**0.042**	0.352
NAc_R	360 (64)	387 (77)	268 (50)		0.044	**0.008**	0.295		0.248	**0.046**	0.390

SD, standard deviation; *P*_FDR_, *P* value after FDR correction; PWS, children with Prader-Willi Syndrome; OB, obese children without PWS; NAc, nucleus accumbens; L, left; R, right. Bold, *P*_FDR_ < 0.05. A: Comparison between the PWS and control groups (PWS vs. control). B: Comparison between the PWS and OB groups (PWS vs. OB). C: Comparison between the OB and control groups (OB vs. control).

**Figure 2:**
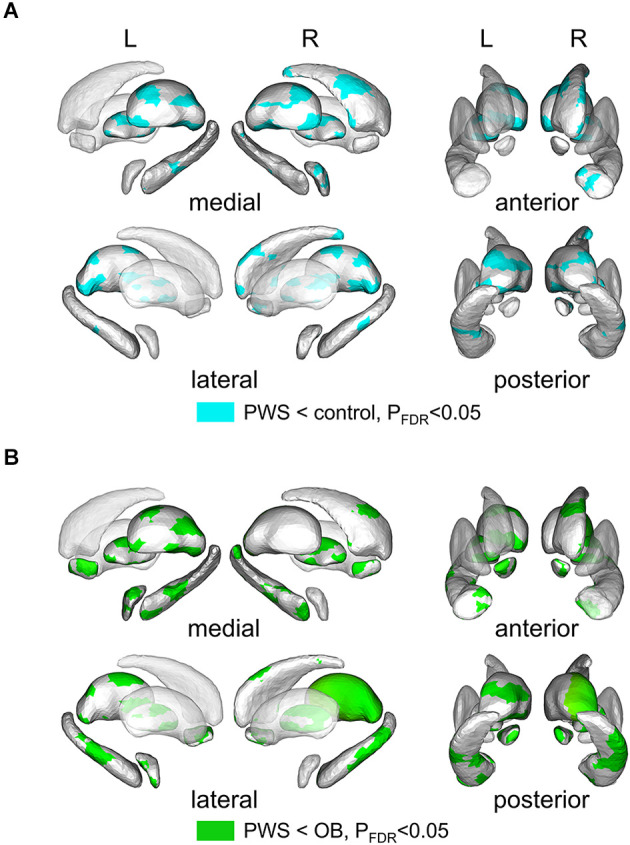
Results of shape analysis in cerebral nuclei, shown from medial, lateral, anterior, and posterior views. **(A)** Atrophic subregions (cyan color) of PWS compared with the control. **(B)** Atrophic subregions (green color) of PWS compared with OB. PWS, children with Prader-Willi Syndrome; OB, obese children without PWS.

Shape analysis of cerebral nuclei showed intergroup differences in subregional shape for each nucleus, which is more sensitive to subtle structural changes than volumetric and VBM analysis (Lu et al., 2016). Figure 2 displays the significantly altered subregions in PWS vs. OB (Figure 2A, color cyan) and PWS vs. control (Figure 2B, color green), with *P*_FDR_ < 0.05 with age, sex, and TIV as covariates. The PWS group showed significant subregional atrophy in the bilateral thalamus, pallidum, hippocampus, amygdala, and NAc, and right caudate compared with OB and control individuals. Although the distribution of altered subregions in these brain nuclei was almost identical in PWS vs. control and PWS vs. OB, the latter group exhibited a wider difference in almost all the atrophic nuclei, except for bilateral caudate and right amygdala. No significant results were found between OB and control subjects.

In addition, since the classification of the boundary voxels was based on intensity distribution of different regions, we calculated the mean intensities of all 14 regions for each subject and compared them between groups (Supplementary Figure 1). No significant differences were found (one-way ANOVA).

### Hypothalamic subunits

The volumetric analysis results of hypothalamic subunits are listed in Table 3. After controlling for age, sex, and TIV, the PWS group had significantly smaller volumes not only in the whole left (control, *P*_FDR_ = 0.022, OB, *P*_FDR_ = 0.020) and whole right (*P*_FDR_ = 0.022) hypothalamus, but also in the subunit parts including bilateral posHyp and infTub, and left a-iHyp, compared with control group (*P*_FDR_ < 0.05) and OB group (*P*_FDR_ < 0.05). In right supTub, a significant difference existed only between the PWS and control groups (*P*_FDR_ = 0.036), while in bilateral a-sHyp and right a-iHyp, no differences were found. Additionally, no significant results were found between OB and control subjects.

**Table 3:** Hypothalamic subunit volumes of each group and *P* value of group comparisons.

	**Mean (SD; mm^3^)**				**Uncorrected *P* value**				***P*_FDR_ value**		
**Subunits**	**Control**	**OB**	**PWS**		**A**	**B**	**C**		**A**	**B**	**C**
a-iHyp_L	10.1 (2.9)	10.6 (2.4)	5.8 (2.7)		**0.012**	**0.011**	0.320		**0.022**	**0.022**	0.494
a-iHyp_R	7.7 (2.4)	8.0 (1.4)	7.7 (1.9)		0.415	0.406	0.243		0.415	0.406	0.494
a-sHyp_L	12.0 (3.3)	12.6 (2.8)	9.9 (1.0)		0.153	0.070	0.468		0.200	0.105	0.494
a-sHyp_R	14.3 (3.7)	14.4 (3.9)	13.4 (2.3)		0.350	0.358	0.494		0.382	0.391	0.494
posHyp_L	103.5 (10.3)	102.2 (8.2)	88.9 (9.3)		**0.011**	**0.013**	0.434		**0.022**	**0.022**	0.494
posHyp_R	99.0 (9.8)	95.0 (10.3)	70.8 (12.2)		**0.003**	**0.005**	0.310		**0.022**	**0.020**	0.494
infTub_L	96.7 (16.6)	109.8 (14.2)	67.3 (17.1)		**0.013**	**0.001**	0.161		**0.022**	**0.012**	0.494
infTub_R	99.2 (16.5)	95.6 (12.6)	60.9 (20.6)		**0.009**	**0.007**	0.375		**0.022**	**0.021**	0.494
supTub_L	89.7 (9.0)	89.2 (9.8)	81.0 (10.7)		0.167	0.159	0.478		0.200	0.212	0.494
supTub_R	95.9 (8.9)	86.6 (11.4)	81.0 (9.0)		**0.024**	0.216	0.139		**0.036**	0.259	0.494
Hyp_L	311.4 (32.7)	324.3 (29.1)	252.9 (35.7)		**0.010**	**0.005**	0.329		**0.022**	**0.020**	0.494
Hyp_R	316.1 (35.4)	299.1 (30.1)	233.7 (41.0)		**0.008**	**0.011**	0.286		**0.022**	**0.022**	0.494

SD, standard deviation; *P*_FDR_, *P* value after FDR correction; PWS, children with Prader-Willi Syndrome; OB, obese children without PWS; a-iHyp, anterior-inferior; a-sHyp, anterior–superior; posHyp, posterior; infTub, inferior tubular; supTub, superior tubular; Hyp, hypothalamus; L, left; R, right. Bold, *P*_FDR_ < 0.05. A: Comparison between the PWS and control groups (PWS vs. control). B: Comparison between the PWS and OB groups (PWS vs. OB). C: Comparison between the OB and control groups (OB vs. control).

### Deep cerebellar nuclei

The volumetric analysis results of the DCN are listed in Table 4. After controlling for age, sex and TIV, the PWS group had a significantly smaller volume in all six DCN ROIs than both the control (*P*_FDR_ < 0.001) and OB (*P*_FDR_ < 0.01) groups. No significant results were found between OB and the control after FDR correction.

**Table 4:** Deep cerebellar nuclei volume of each group and *P* value of group comparisons.

	**Mean (SD; mm^3^)**				**Uncorrected *P* value**				***P*_FDR_ value**		
**Nuclei**	**Control**	**OB**	**PWS**		**A**	**B**	**C**		**A**	**B**	**C**
dentate_L	1,458.7 (124.0)	1,373.2 (143.5)	1,202.7 (136.6)		**0.000**	**0.002**	0.037		**0.000**	**0.003**	0.086
dentate_R	1,593.9 (141.9)	1,517.4 (147.3)	1,311.9 (152.4)		**0.000**	**0.001**	0.067		**0.000**	**0.002**	0.086
interposed_L	197.2 (16.5)	188.1 (18.7)	166.3 (14.4)		**0.000**	**0.001**	0.072		**0.000**	**0.002**	0.086
interposed_R	210.1 (19.7)	201.2 (18.3)	176.3 (15.6)		**0.000**	**0.000**	0.091		**0.000**	**0.002**	0.091
fastigial_L	36.0 (3.5)	34.2 (2.9)	31.6 (2.4)		**0.000**	**0.008**	0.050		**0.000**	**0.008**	0.086
fastigial_R	43.1 (4.4)	40.8 (3.3)	37.4 (3.1)		**0.000**	**0.005**	0.042		**0.000**	**0.006**	0.086

SD, standard deviation; *P*_FDR_, *P* value after FDR correction; PWS, recruited children with Prader-Willi Syndrome; OB, recruited obese children without PWS; L, left; R, right. Bold, *P*_FDR_ < 0.05. A: Comparison between the PWS and control groups (PWS vs. control). B: Comparison between the PWS and OB groups (PWS vs. OB). C: Comparison between the OB and control groups (OB vs. control).

It should be noted that the reduction in DCN volume in PWS was mainly due to WMV loss, either than GMV loss because the total WMV was much greater than the total GMV in DCN ROIs from the SUIT atlas (Supplementary Table 1).

Additionally, decreased GMV of the cerebellar cortex was also found in children with PWS compared with the control group using the VBM method (Supplementary Figure 2 and Supplementary Table 2). The detailed VBM analysis method of cerebellar cortical volume is described in Section “Gray matter volume and white matter volume of deep cerebellar nuclei” of Supplementary Materials.

## Discussion

In the current study, we investigated alterations in brain nuclei in children with PWS and obesity with systematic and combined methods, including shape analysis for cerebral nuclei, automated segmentation methods for the hypothalamus, and VBM for DCN. The results demonstrated that PWS individuals showed significant atrophy in the cerebral nuclei, hypothalamus, and cerebellum compared with control and OB subjects. We also found no significant difference in these brain nuclei between OB and control children. Our results suggest that although PWS and obesity share predominant clinical manifestations (e.g., overweight and hyperphagia) and similar alterations in cortical volume (Xu et al., 2017), impairment in brain nuclei may be very different. Thus, our findings indicate that alterations in brain nuclei might be associated with eating disorders, cognitive disability, and endocrine abnormalities that characterize PWS. Additionally, alterations in brain nuclei were found in PWS individuals during childhood, which might be due to the heritability of subcortical structural shape and the influence of imprinted genes (Roshchupkin et al., 2016), while the relationship between manifestations and brain nuclei in obese children still needs further investigation.

In the shape analysis, atrophy was found in the bilateral thalamus, pallidum, hippocampus, amygdala, NAc, and right caudate in individuals with PWS. The amygdala is the vital nucleus connecting the cortex and hypothalamus, which play an important role in the regulation of food intake by learned and motivational cues (Gottfried et al., 2003; Killgore et al., 2003; Kringelbach et al., 2003; Hinton et al., 2006). The amygdala itself possesses steady-state regulation by maintaining the balance between food satisfaction and a high level of insulin that inhibits eating (Areias and Prada, 2015). Animal studies have suggested that rodents with amygdala lesions showed insulin resistance and typical PWS phenotypes, such as hyperphagia, elevated weight, and hyperinsulinemia (King et al., 1994, 1996; Thaler et al., 2012). Therefore, the volumetric abnormality of the amygdala may be a predictor of such dysfunctions.

Previous studies have reported smaller hippocampal volumes in PWS patients than in normal controls (Honea et al., 2012; Lukoshe et al., 2013; Manning et al., 2018). Consistent with these findings, our results further investigated the shape variation in detail, revealing that the variation was mainly contributed by specific subregions, such as the dorsal, ventral, and lateral of field 1/3 of Ammon’s horn and subiculum. Hippocampal-dependent mnemonic functions play a dominant role in feeding behavior through external sensory food-relevant and internal energy-relevant information (Kanoski and Grill, 2017). Neurons in the dorsal hippocampus receive visuospatial information from the cortex, the ventral hippocampus primarily forms a bidirectional modulation of olfactory cues, and the ventral and lateral hippocampus receive gastrointestinal and other visceral information, all of which determine where, when, what, and how much to eat (Webster et al., 1991; De La Rosa-Prieto et al., 2009; Fanselow and Dong, 2010; Kanoski and Grill, 2017). Patients with bilateral hippocampal lesions show deficits in establishing episodic memory, leading to much more frequent eating. Although the mechanism of hippocampal reduction is unclear, one possible explanation is that hormone signal disturbance caused by abnormal genetic expression influences the formation and maintenance of new memories, including synaptic plasticity and neurogenesis (Shanley et al., 2001; During et al., 2003; Diano et al., 2006; Moult and Harvey, 2008).

The neurons in the NAc regulate motivation for feeding behavior and are activated or inhibited by hormones such as glucose-like-protein-1 or leptin, the dysfunction of which results in excessive food intake (Kenny, 2011; Alhadeff et al., 2012). The subregions of the NAc serve distinct functions; for example, the rostral part contains a hedonic hotspot where “liking” is enhanced for food rewards, while the caudal part contains opioid coldspot where positive hedonic impact is suppressed (Castro et al., 2015). Although the mechanism of NAc volumetric alteration is unclear, the differential performance in PWS and OB may suggest that the reduction is caused by genetically induced cytokine and brain metabolites.

It is well known that limbic cortico-striatal-pallido-thalamic loops are involved in emotional cognition, reward-guided decision-making, and goal-related behavior (Burton et al., 2015; Manning and Holland, 2015). The caudate nucleus plays a dominant role in cognitive function, and its dorsolateral part mediates memory processing and consolidation (Grahn et al., 2008; White, 2009). The ventral pallidum mediates the hedonic influence of food rewards and salience of motivational incentives, particularly through the caudal and rostral parts (Castro et al., 2015). In our study, subregional changes in the thalamus, pallidum and caudate nucleus were noted by the shape analysis method, providing finer neuroimaging evidence than previous findings. Honea et al. (2012) and Kim et al. (2020) reported a larger thalamic volume in obese individuals and a smaller white matter volume in PWS; Hayashi et al. (1992) found coagulative necrosis with nerve cell loss scattered throughout the pallidum; and a small GMV in the caudate nucleus was reported in both PWS and obese patients (Ogura et al., 2011; Kim et al., 2020). Although the genesis of volumetric alteration is unclear, our finding that specific subregions in PWS were much smaller than control and OB groups suggests genetically induced impairment may not be compensated by the acquired neuroadaptive reward system.

However, no morphological change was found in the cerebral nuclei of obese children, according to our study. Previous findings on the relationship between cerebral nuclei volume and obesity are inconsistent. Some studies have reported an enlarged amygdala, hippocampus, thalamus, putamen, and pallidus in obese individuals (Widya et al., 2011; Bernardes et al., 2018; Kim et al., 2020). However, Bobb et al. (2014) reported that higher BMI was associated with a lower volume of the amygdala, and a study with a large sample size of 12,087 subjects from the UK Biobank proved that the percentage of total body fat was negatively associated with subcortical GMVs in men, including the thalamus, caudate nucleus, putamen, globus pallidus, hippocampus, and NAc (Dekkers et al., 2019). From these contradictory evidences, we suggest that there might be sophisticated neurobiological interactions between obesity and cerebral nuclei under physiological and pathological conditions.

Hypothalamic dysfunction is well known to be responsible for the majority of PWS phenotypes, such as hyperphagia, hypersomnia, and multiple endocrine abnormalities (Angulo et al., 2015). However, limited by segmentation methods, most of the studies have not found significantly hypothalamic structural abnormality (Honea et al., 2012; Lukoshe et al., 2017). Recently, using the same Hypothalamic Subunits tool as in this manuscript, Brown et al. (2022) reported PWS adults had smaller volumes of whole hypothalamus and its subunits except for right a-iHyp, than that in obese and control adults. Consistent with the results of PWS adults, atrophy of the bilateral hypothalamus in PWS children was found in our study, specifically contributed by the bilateral in fTub andposHyp, right supTub and left a-iHyp subregions. The hypothalamic paraventricular nucleus, dorsomedial nucleus, and lateral hypothalamic area of the superior tubular hypothalamus, as well as the ventromedial nucleus and infundibular nucleus, comprise the feeding pathway, which facilitates or inhibits feeding through sensing cues, including hormone levels (leptin and ghrelin), visceroceptive inputs (such as food taste), olfactory cues, and glucose-sensitive neurons (Saper and Lowell, 2014). Previous studies have reported that large lesions centered in the ventromedial nucleus lead to hyperphagia and obesity, and neurons in the infundibular nucleus, paraventricular nucleus and lateral hypothalamus nucleus are affected in postmortem hypothalamic tissue specimens from PWS patients (Saper and Lowell, 2014; Correa-da-Silva et al., 2021). Meanwhile, no hypothalamic atrophy was found in OB in our study, which is consistent with a previous study with semiautomated segmentation of the hypothalamus of 338 subjects, indicating that hypothalamic volume is not associated with obesity (Thomas et al., 2019). The relationship between volumetric alterations in the hypothalamus and obesity needs to be further investigated.

The cerebellum plays an irreplaceable role in regulating satiety and meal termination, mainly by DCN and lateral-DCN neurons (Simerly and DiLeone, 2021). The dentate nucleus has been demonstrated to participate in a widespread functional network, including the striatum, hypothalamus, thalamus, sensorimotor and associative cortices, associated with cognition, learning, and sensorimotor coordination (Habas, 2010). The interposed nucleus assists the motor thalamus, mediates eye movement, and responds to tactile stimulation (Judd et al., 2021). The fastigial nucleus receives histaminergic and orexinergic afferents from the hypothalamus and directly projects to the hypothalamus, visceral-related nuclei/regions in the medullary reticular formations and the limbic system to regulate feeding and emotion (Zhang et al., 2016). The dysfunction of DCN is greatly associated with the phenotype of PWS. Low et al. (2021) observed a difference in DCN between individuals with PWS and control participants with fMRI responding to food images. Moreover, mice with a knockout of the *Fmr1* gene showed a reduced volume of DCN and displayed the restricted, repetitive behaviors characteristic of PWS (Ellegood et al., 2010; Wilkes and Lewis, 2018). Meanwhile, as we know, no studies have been reported about DCN alterations in obese children. Consistent with previous studies, we reported a decreased volume of DCN in children with PWS, which was not found in OB. Our findings suggested that the dysfunction of DCN contributed to the developmental behavioral characteristics in PWS and provided evidence that DCN plays an important role in regulating satiety and meal termination in terms of genetics.

There are several limitations that need to be discussed here. First, even though the sample size was small in our study, leading sex and age were not perfectly matched, it had sufficient power to find between-group differences. Meanwhile, as a rare disease, large age ranges and small sample sizes are acceptable in PWS neuroimaging studies (Manning et al., 2018; Kim et al., 2020). Second, structural change in the two major PWS subtypes was not investigated because they do not exhibit clinical differences, except for hypopigmentation (Butler, 1990). Third, the slice number was fixed to 160 slices for all subjects, which was an alternative acquisition mode to the fixed slice thickness, causing the slice thickness to vary with the field of view in z direction. Though all images were resampled to the same voxel size before segmentation, the variation of slice thickness might have potential impact on the precision of segmentation. Fourth, Hypothalamic Subunits tool has not been widely validated on pediatric analysis, except for a recent preprint which was conducted by Voldsbekk et al. (2022) on young samples (age range 5–21 years). Although the quality control procedure was performed manually by an experienced radiologist for every subject in our study, the validation needs to be further investigated. Fifth, unlike subcortical nuclei and hypothalamus, the volume of DCN was calculated by adding total GMV and WMV within each ROI. As discussed previously, VBM methods have limitations in brain nuclei, though we have used the SUIT atlas for better registration. Finally, no significant results were found between OB and the control. Thus, the meaning of different kinds of changes in adult obesity could not be explained by our current study. In the future, the relationship between genetic subtypes and structural change could be investigated to examine the influence of heritable differences involving PWS on brain nuclei.

## Data Availability Statement

The raw data supporting the conclusions of this article will be made available by the authors, without undue reservation.

## Ethics Statement

The studies involving human participants were reviewed and approved by Institutional Review Board at the University of Florida. Written informed consent to participate in this study was provided by the participants’ legal guardian/next of kin.

## Author Contributions

NW and MX conceived the study, performed the formal analysis, and wrote the original draft. NW pursued the implementation of the methodology, conducted the data processing and experiments, generated and validated the results. HY conducted quality control of raw data and the segmentation. All authors contributed to the article and approved the submitted version.

## Funding

The study was supported by the Capital’s Funds for Health Improvement and Research (2020-4-7073), Capital Foundation of Medical Development, and Open access publication fees were supported by Capital Medical University.

## Footnotes

^1^ https://www.cdc.gov/growthcharts/percentile_datafiles.htm

^2^ https://surfer.nmr.mgh.harvard.edu/fswiki/HypothalamicSubunits

^3^ http://www.fil.ion.ucl.ac.uk/spm/software/spm12

^4^ https://github.com/jdiedrichsen/suit

^5^ http://www.nitrc.org/projects/gretna
