# Supplementary material for: Alteration of brain nuclei in obese children with and without Prader-Willi syndrome
Source: Front Neuroinform. 2022 Nov 18;16:1032636. doi: 10.3389/fninf.2022.1032636 (PMC9716021; doi:10.3389/fninf.2022.1032636)
Supplement: Supplementary file 1 [file Data_Sheet_1.docx]

Supplementary Material

# Insensity Distribution of 14 cerebral nuclei of groups

Supplementary Figure 1. Intensity distribution of cerebral nuclei of control, obesity and PWS groups. No significant differences were found between groups (one-way ANOVA, P_FDR_ > 0.05).


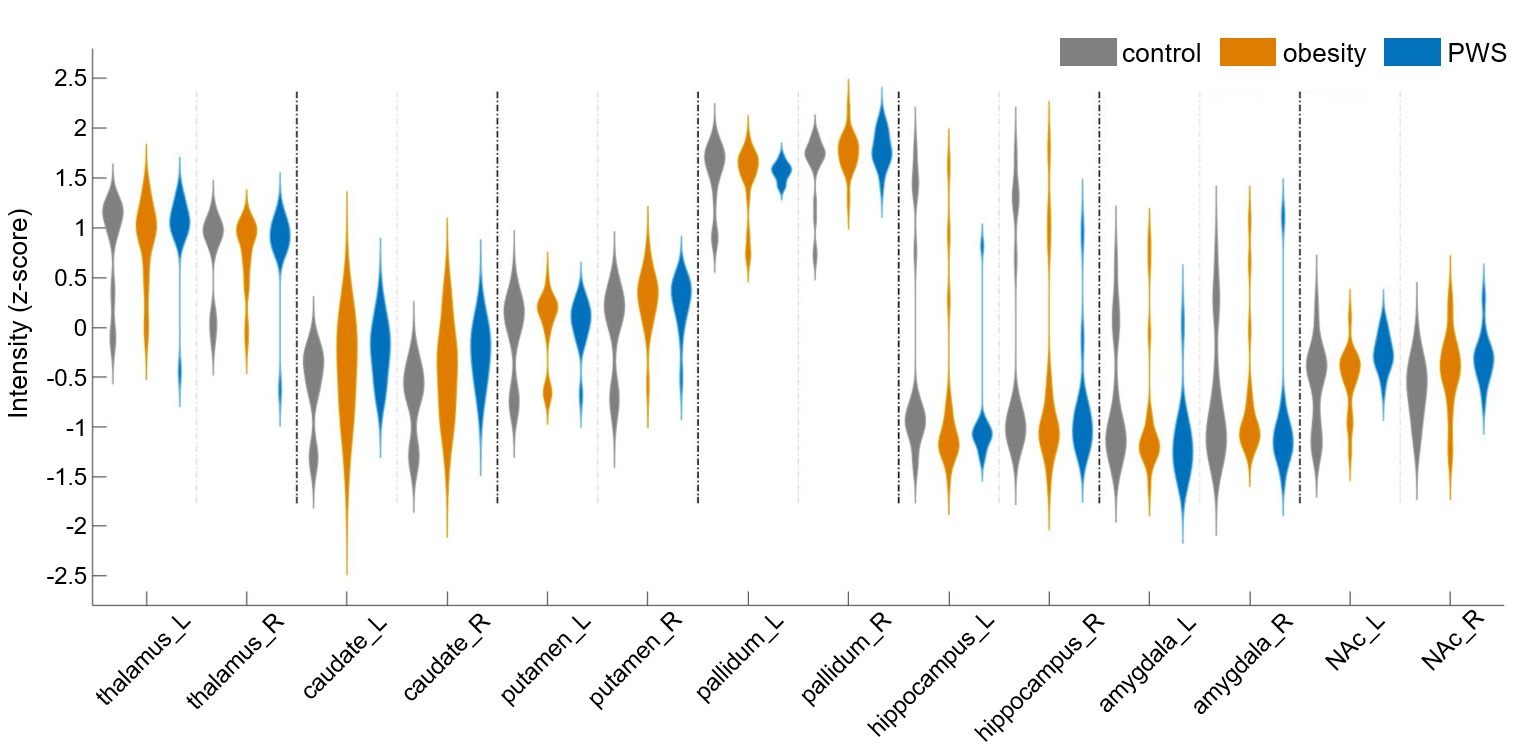


# Gray matter volume and white matter volume of deep cerebellar nuclei

The results of gray matter volume (GMV) and white matter volume (WMV) analysis of DCN are listed in Supplementary Table 1. After controlling for age, sex and TIV, the PWS group had significantly smaller WMVs in all 6 DCN ROIs (bilateral dentate, interposed and fastigial nuclei) than both the control and OB groups (P value after FDR correction (P_FDR_) < 0.05). No significant results were found in WMV between the OB and control groups, and no results were found in GMV between the three groups.

Supplementary Table 1. GMV and WMV of deep cerebellar nuclei for each group and P value of group comparisons

|  | Mean (SD) (mm^3^) | | | | | |  | P_FDR_ value | | |
| --- | --- | --- | --- | --- | --- | --- | --- | --- | --- | --- |
| Nuclei | Control | | OB | | PWS | |  | A | B | C |
| GMV | | | | | | | | | | |
| Dentate_L | 113.2 | (27.4) | 98.7 | (26.7) | 95.5 | (20.0) |  | 0.094 | 0.419 | 0.262 |
| Dentate_R | 113.9 | (20.1) | 108.4 | (27.7) | 99.1 | (24.3) |  | 0.094 | 0.364 | 0.262 |
| Interposed_L | 13.2 | (4.8) | 11.7 | (5.2) | 11.2 | (3.8) |  | 0.156 | 0.419 | 0.262 |
| Interposed_R | 15.5 | (6.1) | 14.0 | (7.4) | 11.6 | (3.9) |  | 0.094 | 0.364 | 0.262 |
| Fastigial_R | 5.4 | (1.5) | 6.3 | (2.4) | 6.1 | (1.7) |  | 0.156 | 0.419 | 0.262 |
| Fastigial_R | 5.0 | (1.4) | 5.4 | (0.8) | 4.9 | (1.0) |  | 0.392 | 0.364 | 0.262 |
| WMV | | | | | | | | | | |
| Dentate_L | 1345.4 | (125.7) | 1274.6 | (133.4) | 1107.2 | (145.5) |  | **0.000** | **0.003** | 0.117 |
| Dentate_R | 1479.9 | (143.2) | 1409.0 | (142.1) | 1212.8 | (164.7) |  | **0.000** | **0.002** | 0.117 |
| Interposed_L | 183.9 | (15.5) | 176.4 | (18.0) | 155.1 | (15.0) |  | **0.000** | **0.002** | 0.121 |
| Interposed_R | 194.6 | (21.5) | 187.2 | (18.6) | 164.7 | (15.6) |  | **0.000** | **0.002** | 0.146 |
| Fastigial_L | 30.7 | (3.6) | 27.9 | (3.6) | 25.5 | (2.6) |  | **0.000** | **0.028** | 0.078 |
| Fastigial_R | 38.1 | (4.5) | 35.4 | (3.3) | 32.5 | (2.9) |  | **0.001** | **0.014** | 0.078 |

SD, standard deviation; P_FDR_, P value after FDR correction; GMV, gray matter volume; WMV, white matter volume; PWS, recruited children with Prader-Willi Syndrome; OB, recruited obese children without PWS; L, left; R, right. Bold, P_FDR_ < 0.05.

A: Comparison between the PWS and control groups (PWS vs. control).

B: Comparison between the PWS and OB groups (PWS vs. OB).

C: Comparison between the OB and control groups (OB vs. control).

# Voxel-based morphometry of cerebellar cortical volume

VBM analysis was used to compare cerebellar GMV by calculating the modulated gray matter tissue probabilistic map. First, VBM processing was conducted with CAT12 (r1907) in SPM (http://www.fil.ion.ucl.ac.uk/spm/software/spm12), including preprocessing, tissue segmentation, spatial registration to Montreal Neurological Institute (MNI) space, modulation and smoothing with a 6 mm FWHM Gaussian kernel (Gaser et al., 2022). Second, to compare cerebellar gray matter intensity among the three groups, a region of interest mask encompassing the entire cerebellum was created. The cerebellum was defined by a validated probabilistic atlas of the human cerebellum available in the SUIT toolbox (https://github.com/jdiedrichsen/suit) (Diedrichsen et al., 2009). GMV was masked with a threshold of 0.25. Finally, statistical analysis was performed with the voxel-wise general linear model (GLM). Significant clusters were formed by employing threshold-free cluster enhancement (TFCE) via 5000 permutations. Clusters with P_TFCE_ value < 0.05 and size > 50 voxels were considered significant results. The contrast was set for PWS vs. OB, PWS vs. control, OB vs. control, with total intracranial volume, age and sex as covariates.

The results of cerebellar VBM analysis are shown in Supplementary Figure 1, and their detailed information is listed in Supplementary Table 2. After controlling for age, sex and TIV, the GMV in the bilateral Crus I/II of the PWS group was significantly smaller than that of the control group (P < 0.05 with TFCE correction). No differences were found between PWS-OB and OB-control comparisons.

Supplementary Figure 2. Results of cerebellar VBM analysis. GMV of the PWS group was significantly smaller than that of the control in Crus I/II (labeled with blue color, P_TFCE_ < 0.05)


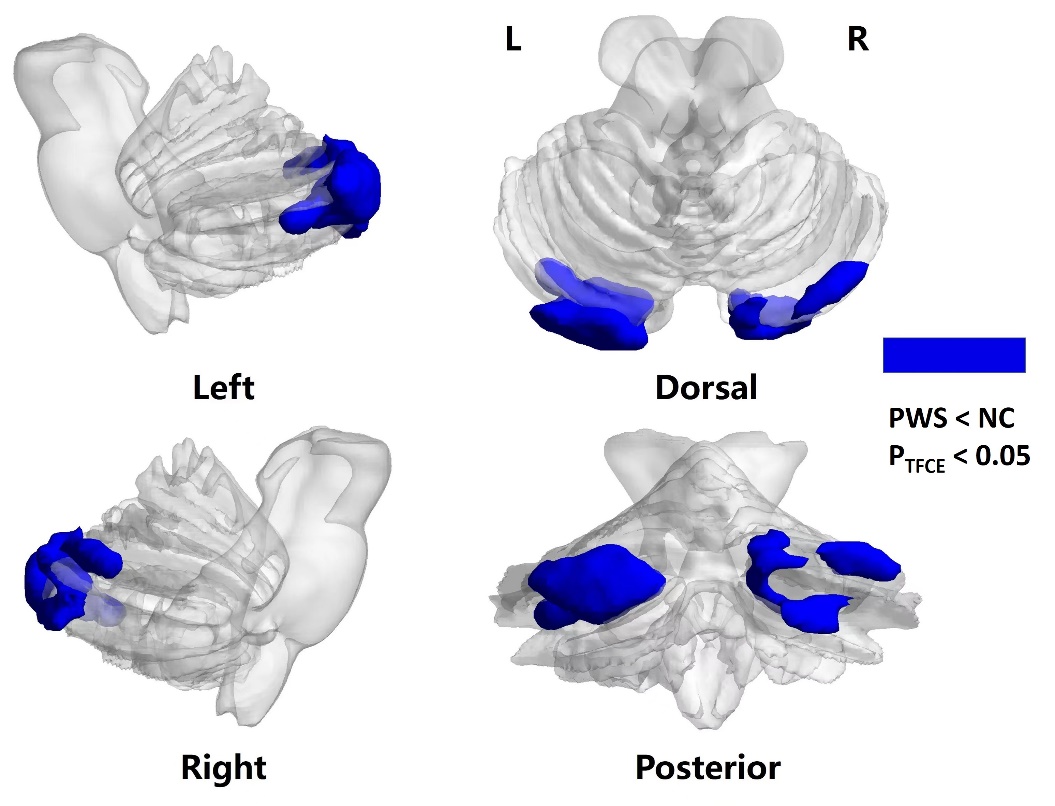


Supplementary Table 2. Cerebellar subregions exhibiting decreased GMV in PWS compared with control

| Cluster | Number of Voxels | Region | MNI Coordinate | | | P_TFCE_ | GMV (Mean (SD) mm^3^) | | |
| --- | --- | --- | --- | --- | --- | --- | --- | --- | --- |
|  |  |  | X | Y | Z |  | control | OB | PWS |
| 1 | 723 | Left  Crus I/II | -24.0 | -82.5 | -31.5 | 0.006 | 1433.6  (170.5) | 1305.8  (177.2) | 1179.6  (162.1) |
| 2 | 588 | Right  Crus I/II | 25.5 | -81.0 | -30.0 | 0.016 | 1124.68  (134.4) | 1060.2  (149.4) | 926.9  (162.9) |

SD, standard deviation; P_TFCE_ was used for VBM analysis of PWS vs. control after TFCE correction. GMV, gray matter volume; PWS, recruited children with Prader-Willi Syndrome; OB, recruited obese children without PWS.
